# A secure and highly efficient blockchain PBFT consensus algorithm for microgrid power trading

**DOI:** 10.1038/s41598-024-58505-w

**Published:** 2024-04-09

**Authors:** Zhongyuan Yao, Yonghao Fang, Heng Pan, Xiangyang Wang, Xueming Si

**Affiliations:** 1 Frontier Information Technology Research Institute, Zhongyuan University of Technology, Zhengzhou, 450007 China; 2 Henan International Joint Laboratory of Blockchain and Data Sharing, Zhengzhou, 450007 China

**Keywords:** Blockchain, Distributed energy trading, Consensus algorithm, Spectral clustering, Zero-knowledge proof, Computer science, Information technology

## Abstract

There are a series of challenges in microgrid transactions, and blockchain technology holds the promise of addressing these challenges. However, with the increasing number of users in microgrid transactions, existing blockchain systems may struggle to meet the growing demands for transactions. Therefore, this paper proposes an efficient and secure blockchain consensus algorithm designed to meet the demands of large-scale microgrid electricity transactions. The algorithm begins by utilizing a Spectral clustering algorithm to partition the blockchain network into different lower-level consensus set based on the transaction characteristics of nodes. Subsequently, a dual-layer consensus process is employed to enhance the efficiency of consensus. Additionally, we have designed a secure consensus set leader election strategy to promptly identify leaders with excellent performance. Finally, we have introduced an authentication method that combines zero-knowledge proofs and key sharing to further mitigate the risk of malicious nodes participating in the consensus. Theoretical analysis indicates that our proposed consensus algorithm, incorporating multiple layers of security measures, effectively withstands blockchain attacks such as denial of service. Simulation experiment results demonstrate that our algorithm outperforms similar blockchain algorithms significantly in terms of communication overhead, consensus latency, and throughput.

## Introduction

Microgrids^1–3^ play a pivotal role in enhancing energy sustainability, ensuring energy security, improving grid stability, enhancing energy efficiency, and promoting environmental sustainability. They are crucial for realizing the vision of sustainable energy^4,5^. According to a recent research report published by the international market research firm Markets and Markets, the global microgrid market was estimated to be approximately $24.6 billion in 2021, and it is projected to increase to $42.3 billion by 2026, with a compound annual growth rate (CAGR) of 11.4% during this period. As microgrid energy technologies continue to mature and energy consumers actively engage in microgrid electricity transactions^6–8^, new opportunities and challenges have emerged in this field. When energy consumers actively participate in microgrid electricity transactions, the opportunities lie in enhancing energy independence and promoting sustainable development. However, this also brings about regulatory complexities^9^, interoperability requirements^10^, data privacy concerns^11,12^, and challenges related to supply-demand balance^13^.

Research on microgrid transactions based on blockchain technology has attracted the attention of many researchers^14,15^. Reference^16^ uses blockchain technology as a distributed data storage technology to deal with some problems caused by the continuous expansion of the scale of distributed power generation microgrids, and proposes a two-layer framework for multi-microgrid energy transactions based on blockchain to facilitate transactions. In research^17^, it explores the possibility of developing blockchain-enabled smart microgrids (BSMG). It aims to build a conceptual framework of BSMG, including the transaction protocols and process flows. Reference^18^ leverages the advantages of blockchain in proposing a secure energy trading platform for all parties involved. Coupled with certificateless signcryption, an immutable energy trading market is designed, and its use case is applicable in smart cities. Blockchain technology^19–21^ is an emerging technology that has garnered significant attention due to its decentralized and trustless characteristics. With the help of smart contracts, blockchain technology can facilitate automatic execution of transactions while ensuring transparency in transaction information and immutability of transaction data. The integration of blockchain technology with microgrid transactions holds the potential to address the opportunities and challenges mentioned above to some extent. In terms of opportunities, blockchain technology can enhance energy autonomy within microgrids, promote sustainable energy development, and support decentralized energy markets, allowing stakeholders to engage in autonomous transactions. Additionally, it can provide more transparent transaction records, making it easier for regulatory authorities to monitor energy transactions^22–24^, promote interoperability between different microgrid systems, and safeguard user energy data privacy^25,26^. However, microgrid transactions are typically characterized by high-frequency and low-value transactions, which pose some challenges when integrating with blockchain technology. One of the key challenges involves the consensus algorithm of the blockchain^27–29^, which is tasked with ensuring transaction validation and their addition to the blockchain. Inefficient consensus algorithms can lead to a series of issues. Firstly, it can limit the number of transactions that can be processed per second, potentially leading to transaction congestion, especially in the case of high-frequency transactions. Secondly, it can prolong transaction confirmation times, which may affect scenarios in microgrids where real-time transactions are required. Additionally, inefficient consensus algorithms may not align with the sustainability goals of microgrids, leading to unnecessary energy consumption. Most importantly, inefficient consensus algorithms may increase the risk of the system being vulnerable to attacks such as double-spending attacks or denial-of-service attacks, as attackers can easily push the network to its limits, reducing performance, or causing system failures. Therefore, when integrating blockchain technology with microgrid transactions, choosing an efficient and secure blockchain consensus algorithm is of paramount importance. Efficient consensus algorithms contribute to improving the performance and efficiency of blockchain-based microgrid transaction systems, making them more suitable for handling high-frequency, small-value transactions, and providing users with superior services.

PBFT (practical byzantine fault tolerance)^30^ is a highly suitable blockchain consensus algorithm for microgrid transactions, offering several advantages. These advantages include providing both real-time and eventual consistency, adaptability to high-frequency small-scale transactions, high security, and resilience against Byzantine faults. These features enhance the trustworthiness and resistance to attacks of microgrid transaction systems. However, it should be noted that as the number of nodes within the microgrid increases, the communication overhead of PBFT also escalates significantly, which can potentially lead to a decrease in blockchain system performance. To address the aforementioned challenges, this study introduces a sharding-based Practical Byzantine Fault Tolerance algorithm tailored for microgrid transactions, referred to as S-PBFT (Practical Byzantine Fault Tolerance Based on Sharding), building upon the PBFT consensus algorithm. S-PBFT is a blockchain consensus algorithm that is highly suitable for microgrid transactions, offering a comprehensive solution to various challenges encountered in microgrid transactions. S-PBFT employs a comprehensive authentication scheme that combines the Schnorr protocol with the Diffie-Hellman (DH) key exchange algorithm to authenticate node identities while safeguarding their privacy information. Furthermore, by Spectral clustering nodes based on their historical transaction characteristics and involving these clusters as lower-level consensus sets in the consensus process, it enhances the reliability of consensus. The election strategy for consensus set leaders is also designed based on the historical transaction behavior of nodes, thereby reducing the involvement of Byzantine nodes. Finally, S-PBFT employs a dual-layer consensus process where the lower-level consensus set performs local consensus, while the upper-level consensus set conducts global consensus on the consensus results. This approach reduces the burden on network communication, reduces the time required for consensus, and enhances the system's throughput and security. The goal of S-PBFT is to provide an efficient and secure consensus algorithm to meet the demands of large-scale microgrid electricity transactions while ensuring real-time responsiveness and adaptability of the system. The main contributions of this research can be summarized as follows:

1. Introduced an efficient and secure consensus model tailored for blockchain-based microgrid electricity trading scenarios. This model incorporates a layered architecture consisting of several critical phases, including registration, preparation, two-tier consensus, and leader election updates;
2. Introduced the S-PBFT consensus algorithm, comprising the collaborative efforts of multiple sub-algorithms, such as the key negotiation algorithm based on zero-knowledge identity authentication, the consensus set partitioning algorithm based on Spectral clustering, the consensus set leader election algorithm, and the dual-layer consensus process;
3. Performed a detailed security analysis of the proposed consensus algorithm and evaluated the algorithm’s performance through comparative experiments. The experimental results demonstrate that, compared to existing algorithms, S-PBFT outperforms in the context of blockchain-based microgrid electricity trading scenarios.

The organizational structure of this paper is as follows: Sect. “Related work and preliminary knowledge” introduces related work and preliminary knowledge. Sections “Blockchain-based distributed power transaction consensus model” and “Design and implementation of S-PBFT consensus algorithm” explain the proposed methodology of this research. Section “Security analysis” discusses the security aspects of the study. Section “Performance analysis” discusses the experimental setup and simulation results. The conclusion and future directions are provided in Sect. "Conclusion".

## Related work and preliminary knowledge

### Related work

Currently, research on blockchain consensus algorithms for microgrid power trading is relatively limited. Given that nodes in microgrids are susceptible to attacks and may exhibit malicious behavior, consensus algorithms with BFT (Byzantine Fault Tolerance) capabilities are more suitable for microgrid power trading scenarios compared to other algorithms. The PBFT (Practical Byzantine Fault Tolerance) algorithm is renowned for its real-time performance, eventual consistency, high security, and resistance to attacks, making it highly suitable for microgrid transaction scenarios. However, as the number of nodes within the network increases, the performance of the PBFT algorithm rapidly deteriorates. Therefore, a significant number of researchers have made improvements to the PBFT algorithm to address this issue.

Reference^31^ introduced the ABC-GSPBFT algorithm (artificial bee colony-group scoring PBFT), which incorporates a group scoring mechanism and artificial bee colony optimization into the consensus process. This algorithm employs an artificial bee colony algorithm to preselect reliable nodes for consensus, utilizes a group scoring mechanism to dynamically update consensus nodes, simplifies the PBFT algorithm’s submission phase, reduces consensus latency and communication overhead, and enhances the dynamic performance and consensus efficiency of flight data sharing. Reference^32^ introduced the ULS-PBFT (ultra-low storage PBFT) consensus, which minimizes storage overhead by hierarchically grouping nodes to limit storage costs within each group. This approach enables PBFT-based blockchain systems to be deployed in large-scale network scenarios. Reference^33^ divided large-scale network nodes into different consensus groups based on response time and conducted group consensus. They also designed a related credit model and voting mechanism for dynamic updates of consensus nodes. The proposed P-PBFT algorithm (Placeholder for P-PBFT) effectively addresses issues such as high latency, high system overhead, and support for smaller-scale applications in the context of combining pharmaceutical traceability with blockchain technology. Reference^34^ introduces the probability language term set with confidence intervals (PLTS-CI) for the practical Byzantine Fault Tolerance (PBFT) consensus mechanism. This terminology is used to express uncertain and complex voting information among nodes during leader election. The approach employs sophisticated decision attitudes to select the highest-scoring nodes, thereby effectively improving the efficiency of achieving consensus. Reference^35^ addresses the limitations of delay, transaction frequency restrictions, and severe block congestion in the vehicular ad-hoc network consensus process. It introduces a fast and intelligent consensus mechanism called R-PBFT. Experimental results demonstrate that R-PBFT outperforms state-of-the-art methods and is resilient to various attacks. Reference^36^ presents an improved blockchain consensus algorithm using a genetic algorithm-based approach. It designs fitness functions for blockchain nodes and applies a genetic algorithm to iteratively generate consensus node groups with superior performance metrics. By selecting high-performance nodes, the algorithm enhances the speed and efficiency of consensus, block generation, and computation while maintaining reliability with a limited number of consensus nodes. Reference^37^ introduces a main node selection method based on node reputation evaluation. It selects main nodes according to their reputation values, and the use of node reputation in determining node identities reduces the likelihood of main nodes being erroneous nodes. This approach results in fewer communication instances and lowers communication overhead during the process. Reference^38^ introduces the hierarchical practical byzantine fault tolerance (H-PBFT) consensus algorithm. It divides the consensus of the entire network into multiple sub-layers, reducing communication complexity and enhancing fault tolerance. This improvement enhances the scalability of the blockchain, enabling it to efficiently support large-scale nodes for transmission and communication. Reference^39^ proposes a lightweight blockchain algorithm based on secure practical byzantine fault tolerance (PBFT) consensus for healthcare applications, considering the limited computational power of IoT devices in IoT environments. It uses a feature trust model and verifiable random functions (VRF) to randomly select a primary node from a group of trusted consensus nodes, improving consensus efficiency and system security. Reference^40^ introduces a consensus mechanism that combines PoS (proof of stake) with PBFT (practical byzantine fault tolerance). While maintaining high performance, it effectively handles dishonest nodes. The model uses trust scores and reward mechanisms as key components of the block validation and ordering process to incentivize honest behavior. Performance is evaluated using an analysis model of a semi-Markov process, demonstrating that consensus efficiency can be maintained even in scenarios with a high likelihood of dishonest node behavior. The above-mentioned research has made significant advancements, but these optimization methods have yielded relatively modest improvements in performance, making it challenging to meet the high throughput requirements of energy transactions in microgrid environments. Therefore, there is an urgent need for an efficient and secure blockchain consensus algorithm to meet the characteristics of high-frequency transactions in microgrids.

### Preliminary knowledge

#### Blockchain technology

Blockchain technology is essentially built upon microgrids, decentralization, secure encryption, and consensus mechanisms. Its primary objective is to establish a trustworthy, transparent, and tamper-proof data recording and transaction system. It provides new solutions for applications across various fields, fundamentally altering the operation of traditional centralized systems. Based on permissibility, blockchain can be categorized into two types: permissioned blockchains and permissionless blockchains. These two types of blockchains have different advantages and uses in microgrids, and Table 1 compares the characteristics of these two types of blockchains.

**Table 1:** Comparison between two types of blockchains.

Blockchain type	Permissioned blockchains	Permissionless blockchains
Node permission	Requires authorized nodes	Anyone can participate
Identity verification	Nodes typically require verification	No identity verification required
Participation in consensus	Restricted participants	Open participation
Control	Managed by specific entity or organization	No specific entity or organization in control
Privacy	Emphasizes privacy and permission control	Emphasizes openness and transparency
Consensus algorithm	Uses efficient consensus algorithms	Uses energy-intensive consensus algorithms

Permissioned blockchains offer several advantages in microgrids, including more flexible permission management, enhanced data privacy protection, and ease of meeting regulatory compliance requirements. For example, microgrid systems can use permissioned blockchains to ensure that only authorized participants can manage the flow of electricity, thereby enhancing security and controllability. Furthermore, permissioned blockchains can offer more robust data privacy protection mechanisms, ensuring that sensitive information remains inaccessible to unauthorized individuals. Most importantly, permissioned blockchains allow microgrid systems to more easily comply with regulatory requirements because they offer stronger authentication and regulatory tools. Applying permissioned blockchains to microgrids also helps simplify supply chain management, increase data transparency, and reduce the complexity of energy transactions, thereby bringing higher reliability and efficiency to microgrid systems. Therefore, this paper constructs a blockchain-based microgrid transaction system based on permissioned blockchains.

#### PBFT consensus algorithm

**Figure 1:**
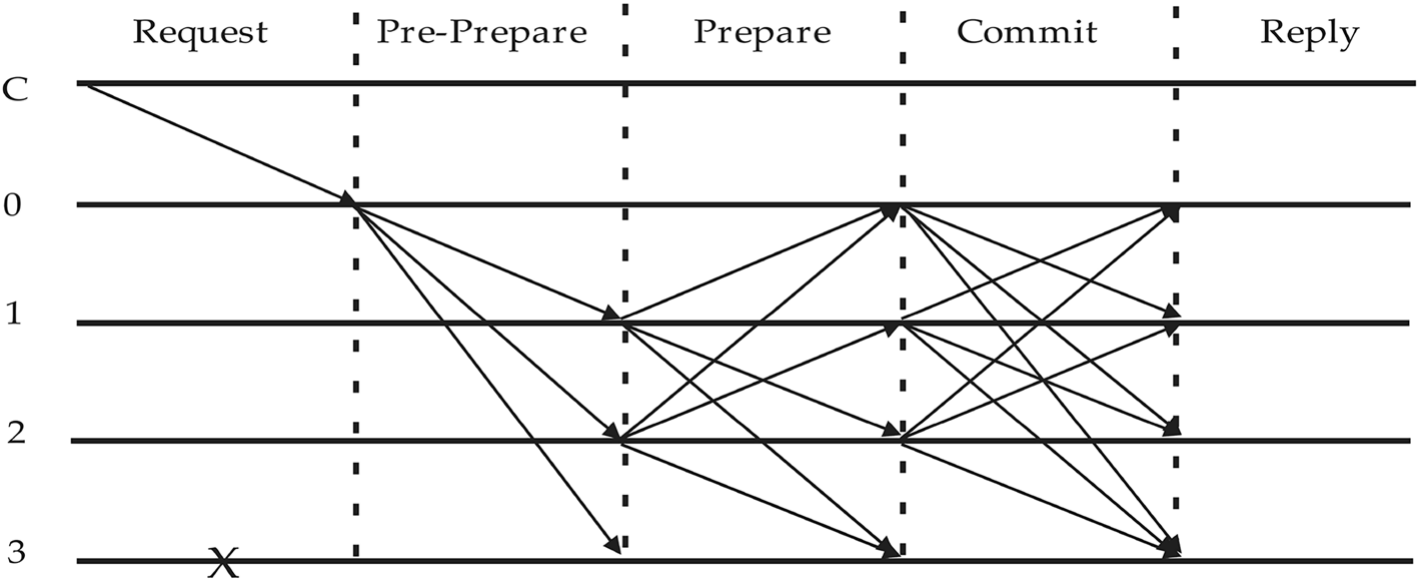
PBFT consensus process.

The PBFT algorithm is widely adopted in microgrid computing. Its primary objective is to ensure that even in the presence of node failures or malicious behavior within the microgrid system, the system can continue to operate smoothly and achieve consensus. PBFT has the capability to tolerate (Byzantine) node failures or malicious behavior, allowing for as many as *N/3* nodes in the microgrid system to be Byzantine nodes, where N is the total number of nodes participating in the consensus process. The PBFT algorithm consists of a consensus protocol, a checkpoint protocol, and a view-change protocol. The consensus protocol is used to ensure that nodes agree on the order of transactions, the checkpoint protocol is used to maintain a snapshot of the system's state, and the view-change protocol is used to handle failures of Byzantine nodes. The consensus process of PBFT is illustrated in Fig. 1.

#### Zero-knowledge identity authentication

The Schnorr protocol is a cryptographic protocol and zero-knowledge proof mechanism used for digital signatures and key exchange. It was proposed by Klaus Schnorr in 1991. The Schnorr protocol is known for not only its high security but also its excellent efficiency, flexibility, and privacy protection features. The Schnorr protocol allows the prover to claim knowledge of a certain key value *x* without revealing *x* itself to the verifier.

In this paper, the authentication scheme we use combines the Schnorr zero-knowledge identity proof and the Diffie-Hellman (DH) key exchange algorithm. The objective of this scheme is to ensure that nodes can verify the validity of other nodes’ identities without revealing private data. This scheme ensures privacy protection for identity verification based on public parameter information on the blockchain ledger. In terms of security, the authentication scheme relies on the discrete logarithm problem, making it highly secure. This comprehensive authentication scheme plays a crucial role in safeguarding privacy and ensuring security. It is expected to find widespread application in various scenarios.

## Blockchain-based distributed power transaction consensus model

### Model description

**Figure 2:**
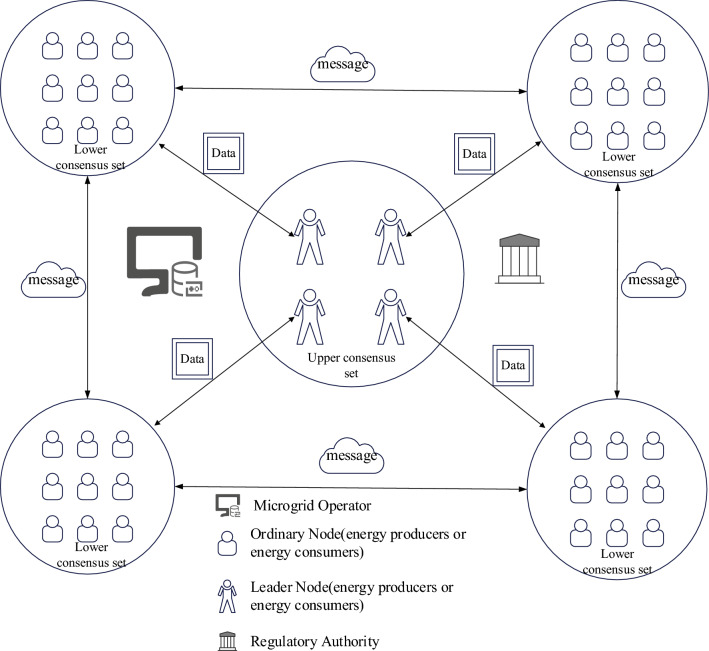
Blockchain-based microgrid power trading consensus model.

The blockchain-based microgrid power trading consensus model proposed in this paper is illustrated in Fig. 2. The consensus model primarily consists of four entities: the microgrid operator, energy producers, energy consumers, and regulatory authorities. Entities can communicate with each other, with energy producers and energy consumers considered as consensus nodes within the blockchain network. When energy producers and consumers request transactions, they must initiate transaction requests with the microgrid operator. After the transaction is completed, the transaction information stored in the blockchain is updated accordingly.

#### Microgrid operator

The microgrid operator is responsible for overseeing and managing the operation of the microgrid, which includes energy production, distribution, and maintenance. They could be energy suppliers, community organizations, businesses, or government agencies.

#### Energy producers

Energy producers may include solar panels, wind turbines, small-scale gas generators, etc., responsible for generating electricity within the microgrid.

#### Energy consumers

Energy consumers are the end-users of the microgrid, using energy to meet household or business needs. They may also be energy producers.

#### Regulatory authorities

Government or industry regulatory bodies may need to oversee the compliance of the microgrid trading platform to ensure that transactions adhere to regulations and standards.

### Consensus process

To address the characteristics of high-frequency and small-value electricity transactions within microgrids, this paper introduces a practical Byzantine Fault Tolerance consensus algorithm called S-PBFT. S-PBFT, while maintaining efficiency and security, is capable of supporting large-scale electricity transactions within microgrids. The algorithm primarily consists of four phases, including registration, preparation, consensus, and election value update.

#### Registration phase

In the microgrid electricity trading network, energy producers and consumers are required to meet the eligibility requirements for electricity transactions. Before nodes can join the blockchain network, they must complete the registration process, which includes submitting a registration application to the regulatory authority. The regulatory authority is responsible for reviewing these registration applications to ensure that nodes meet the relevant criteria and regulations. Upon successful review, the microgrid operator will issue a unique identity certificate to the node and initialize the node's information. This identity certificate serves as the node’s unique identity identifier within the blockchain network and plays a crucial role in subsequent identity authentication processes. This way, nodes can securely participate in the consensus and transaction activities within the electric power trading network after successfully passing the identity verification.

#### Preparation phase

In the preparation phase, the first step involves obtaining the wireless network coordinates of the nodes and Spectral clustering them into k lower-level consensus set using a Spectral clustering algorithm. Next, nodes within each consensus set will execute an authentication protocol to ensure the legitimacy of their identities and exchange necessary information with other nodes. Following that, based on the results of the election values, a leader node will be elected within each consensus set, and this leader node will become the leader of the lower-level consensus set and collaborate with other leader nodes to form the upper-level consensus set. This process ensures that in the microgrid power trading network, nodes are effectively organized and categorized, and there is a leader node in each consensus set responsible for managing and coordinating the consensus process. This hierarchical structure helps improve the efficiency and reliability of power trading in the microgrid.

#### Dual-layer consensus process phase

S-PBFT consensus is divided into two parts: the lower-level consensus set and the upper-level consensus set. In the lower-level consensus set, regular nodes reach consensus on the blocks proposed by the leader node of the lower-level consensus set. Once the lower-level consensus is successful, the leader node sends the block to the upper-level consensus set, and then this leader node guides the completion of consensus in the upper-level consensus set. Finally, the blocks achieved through consensus are broadcast by the rest of the nodes in the upper-level consensus set to each lower-level consensus set, where ordinary nodes validate and update their local ledgers. This process ensures the effective propagation of consensus and block validation, ensuring data consistency and security.

#### Election value update phase

Based on the nodes’ transaction behavior during this consensus, the election values are updated. In leader elections, factors such as transaction participation level, transaction capability, energy type preference, and trustworthiness score are typically considered. Based on these factors, nodes can calculate their election values and submit them to the consensus system to participate in leader elections. This helps ensure that the selection of leader nodes is based on the actual performance and contributions of each node, thereby improving the efficiency and reliability of the consensus system.

In S-PBFT consensus, each successful completion of consensus or consensus timeout triggers a view change to enter a new round of consensus. Typically, the consensus system sets a threshold (e.g. *v* rounds of consensus), and when this threshold is reached, it triggers a view change, initiating a new consensus cycle. Before the start of a new cycle, the consensus system typically reapplies the Spectral clustering algorithm to partition nodes into consensus sets. This process ensures that consensus can be achieved within a certain timeframe and number of rounds in the microgrid power trading network. It also allows for view changes under certain conditions to address potential consensus failures or timeouts. Repartitioning the consensus sets helps adapt to changes and dynamics in the network’s nodes, enhancing the robustness and performance of the consensus system.

## Design and implementation of S-PBFT consensus algorithm

To enhance the security and efficiency of blockchain consensus algorithms in microgrid power trading, the proposed S-PBFT consensus algorithm in this paper comprises four sub-algorithms: the authentication protocol, consensus set partitioning, dual-layer consensus process, and consensus set leader election strategy. Firstly, the microgrid operator employs a Spectral clustering algorithm to group users based on their transaction characteristics, dividing the blockchain network into *K* consensus sets. Subsequently, nodes within different consensus sets use an authentication protocol combining zero-knowledge proofs and the DH algorithm to authenticate other nodes, ensuring that only legitimate nodes can participate in the consensus process. Subsequently, a lower-level consensus set is randomly selected to complete the first round of the consensus process. After successful consensus, the leader node submits the consensus result to the upper-level consensus set, completing the second round of global consensus. After the two-level consensus, each node updates the blockchain ledger based on the consensus result. Finally, based on the historical transaction information of users, trusted consensus leaders are elected within the consensus set. These leaders will be responsible for guiding the smooth completion of the consensus process. These sub-algorithms collaborate together to enhance the security and efficiency of microgrid power transactions.

### Key negotiation algorithm based on zero-knowledge identity authentication

The introduction of authentication protocols in consensus networks can provide security, reliability and performance guarantees, ensure the legitimacy and credibility of nodes in the consensus process, and maintain the consistency of the system. In the S-PBFT algorithm, each node has a unique identity. Before a node participates in the consensus process, each node stores its own identity certificate on the blockchain. If a node wants to verify the validity of the identity of other nodes in the network, it can verify it by calling a smart contract. This article uses the Schnorr protocol for identity authentication. The Schnorr protocol uses a concise mathematical proof method that can achieve zero-knowledge proof with less calculation and communication. Select the DH key exchange algorithm for the key agreement mechanism. By using the DH algorithm, multiple nodes can safely generate shared keys without the need to share the keys in advance, thereby supporting secure communication and collaboration in distributed systems. Both Schnorr proof and DH algorithm are based on the discrete logarithm problem, which is very powerful in terms of security and difficult to be cracked or attacked. The specific process is as follows:

1. The transaction regulatory authority randomly selects a prime number *p*, satisfying that *p-1* can be divided by a small prime number *q*. They also select a generator *g*, which is a primitive root modulo *p*. These parameters are then sent to Node *i* and Node *j*.
2. Node *i* calculates the DH algorithm interaction parameter $Yi = g^{a} \bmod p$ based on its private key *a*,where 0 < *a* < *q*. Node *j* similarly computes the DH algorithm interaction parameter $Yj = g^{b} \bmod p$ based on its private key *b*, where 0 < *b* < *q*.
3. Node *i* selects a random number *r*_*1*_ and computes $R1 = {\text{g}}^{r1}$, $z1 = r1 + ac1$, where $c1 = H\left( {r1 \cdot Yi} \right)$ is used for Node *i* ‘s Schnorr zero-knowledge proof. Node* j* selects a parameter *r*_*2*_ and calculates $R2 = {\text{g}}^{r2}$, $z2 = r2 + ac2$, where $c2 = H\left( {r2 \cdot Yj} \right)$ is used for Node *j* 's Schnorr zero-knowledge proof.
4. Node *i* sends the DH algorithm interaction parameters and the Schnorr zero-knowledge proof to Node *j*. Similarly, Node *j* sends the DH algorithm interaction parameters and the Schnorr zero-knowledge proof to Node *i*.
5. Node *i* validates the effectiveness of the Schnorr proof. If the equation $g^{z2} = R2 \cdot Y_{i}^{c2}$ holds true, then the proof is valid. Node *i* computes the shared key $K = Yi^{b} \bmod p$. Node *j* validates the effectiveness of the Schnorr proof. If the equation $g^{z1} = R1 \cdot Y_{i}^{c1}$ holds true, then the proof is valid. Node *i* computes the shared key $K = Yj^{a} \bmod p$.

### Consensus set partitioning based on spectral clustering algorithms

When a sufficient number of users are added to a blockchain network, it becomes crucial for nodes to be divided into different consensus sets to ensure the efficiency of the consensus algorithm. S-PBFT introduces a consensus set leader election mechanism and a consensus set division strategy based on spectral clustering. Through spectral clustering, the blockchain network is divided into different consensus sets, each of which has consistent functionality and equal status in the network. Considering that the PBFT algorithm requires at least 4 nodes to execute, the number of nodes in each consensus set needs to be controlled, that is, *n* ≥ 4. In view of the fact that the PBFT algorithm will significantly increase the network traffic when the number of nodes is large, this article defines the node number threshold as *n* < *N/k* to ensure the efficient operation of the consensus network and reduce the load caused by the PBFT algorithm on the blockchain network.

Spectral clustering facilitates the division of nodes in a microgrid into different electrical subsystems, thereby optimizing and managing the operation of the microgrid. This partitioning refines the consensus process, making it easier for nodes within each subsystem to reach consensus, thus reducing the complexity and communication overhead of the consensus process. Additionally, spectral clustering can be utilized to detect anomalies and faults within nodes of the microgrid, aiding in the timely implementation of measures to maintain the stability and reliability of the microgrid, thereby enhancing the robustness of the consensus algorithm. The spectral clustering process is as follows:

1. Assuming there are *N* nodes in the blockchain network, *V* represents all the nodes in the blockchain network (*x*_*1*_, *x*_*2*_, ..., *x*_*n*_, *n*=|*N*|). Taking *N* nodes as* N* vertices and *E* as the set of edges between vertices, an undirected full graph *G* = (*V*, *E*) can be formed.
2. Define an adjacency matrix $W = \left[ {\alpha \left( {xi,xj} \right)} \right] \in {\mathbb{R}}^{N \times N}$ based on the nodes in the blockchain network, where *x*_*i*_ and *x*_*j*_ are node vectors, and *a(x*_*i*_*, x*_*j*_*)* represents the similarity, computed as follows.
  1 $$ \alpha \left( {xi,xj} \right) = \exp \left( { - \frac{{\left\| {xi - xj} \right\|_{2}^{2} }}{{2\sigma^{2} }}} \right), $$

where σ is the width parameter of the function. A larger value for parameter σ results in higher similarity between nodes. It should be adjusted based on the specific context and requirements.

(3) The degree matrix is a diagonal matrix, and the values on the main line consist of the degrees of the corresponding vertices. Define the degree matrix $D = diag\left( {d1,d2, \cdots ,dn} \right)$, where *d*_*i*_ represents the sum of the weights of all edges connected to any vertex *v*_*i*_, i.e.
  2 $$ di = \sum\limits_{j = 1}^{N} {wij} , $$
  where *w*_*ij*_ represents the edge weight between vertex *v*_*i*_ and vertex *v*_*j*_.
(4) For a graph *G* = (*V*, *E*) with *n* vertices, its Laplacian matrix is defined as $L = D - W$. Calculate the Laplacian matrix $L = D - W$, and subsequently normalize $Lsym = D^{{ - \frac{1}{2}}} LD^{{ - \frac{1}{2}}}$.
(5) Calculate the eigenvectors *f* corresponding to the *k* smallest eigenvalues of *L*_*sym*_.
(6) Normalize the rows of the matrix composed of the individual feature vectors *f* to create the feature matrix $F\in {R}^{N\times k}$.
(7) Apply the k-means clustering algorithm to cluster each row $r1,r2, \cdots ,rn$ in the matrix *F*, resulting in *k* node clusters $A1,A2, \cdots ,Ak$.

Once all nodes within the blockchain network are partitioned into different consensus sets through spectral clustering, the blockchain network will elect reliable leader nodes in each consensus set as consensus nodes for the current consensus process, according to the leader election method outlined in Sect. “Consensus set leader node election”. Once consensus nodes are elected, the consensus set will remain fixed until the end of the current consensus round. If a consensus node withdraws from the consensus set during the consensus period, the node with the second-highest election value, based on the descending order principle outlined in Sect. “Consensus set leader node election”, will be selected as the new consensus node to participate in the consensus. After the current consensus round concludes, all nodes will redivide the consensus sets based on the results of the previous consensus round.

### Consensus set leader node election

In S-PBFT, factors such as transaction participation level, transaction capability, energy source preference, and trustworthiness score are considered as the primary criteria for group leader elections. When selecting the initial leader node, a random node is elected as the leader node. Subsequently, new leader nodes will be elected based on a combination of factors, including the results of each round of transactions. The definitions of these factors are as follows:

#### Definition 1

The transaction participation level, denoted as *B* for a node, is a crucial metric that assesses the level of activity of the node in microgrid power transactions. It depends on the number of transactions in which the node has participated during past trading cycles. Assuming node *I*’s set of transaction participations is $\beta i=\left\{{b}_{1}^{i},{b}_{2}^{i},{b}_{3}^{i},\cdots ,{b}_{t}^{i}\right\}$, where $b_{t}^{i}$ represents the number of transactions node *i* participated in during transaction cycle *t*, the transaction participation level *B* for each node is calculated as shown in Eq. (3):

3 $$ B = \sum\limits_{r = 1}^{t} {\delta \cdot \frac{{b_{r}^{i} }}{{b_{r}^{\max } }}} , $$

where $b_{r}^{\max }$ represents the maximum number of transactions across all nodes in the network during transaction cycle *s*, serving as a measure of the overall network activity. *δ* is a decay coefficient smaller than 1, used to indicate that the influence of previous transactions on nodes gradually diminishes over time.

#### Definition 2

The transaction capability of a node, denoted as *E*, reflects its level of contribution to the operation of the microgrid electricity trading network. In the same trading period, $s_{ij}^{m}$ represents the transaction satisfaction of node *i* with respect to node* j* in the *m*-th transaction within that period, used to measure the satisfaction level between nodes in specific transactions.$er_{ij}^{m}$ represents the estimated amount of electricity traded between node *i* and node *j* when signing a smart contract, while $ep_{ij}^{m}$ represents the actual amount of electricity transferred between node *i* and node *j*. $s_{ij}^{m}$ represents the transaction outcome between node *i* and node *j*, and its value can be obtained from the following Eq. (4):

4 $$ s_{ji}^{m} = \left\{ {\begin{array}{*{20}c} 1 & {er_{ij}^{m} \ge ep_{ij}^{m} } \\ { - 1} & {er_{ij}^{m} < ep_{ij}^{m} } \\ \end{array} } \right.. $$

If $s_{ij}^{m} = 1$, it indicates that node *i* and node *j* have successfully completed a transaction, otherwise, if $s_{ij}^{m} \ne 1$, it signifies that the transaction between node *i* and node *j* has failed. Node’s transaction capability *E* can be calculated using Eq. (5):

5 $$ Ei = \sum\limits_{r = 1}^{t} {\eta \cdot \sum\limits_{j = 1,j \ne i}^{n} {\sum\limits_{m = 1}^{m} {s_{ij}^{m} } } \cdot er_{ij}^{m} } . $$

In the above description, *η* is a decay factor less than 1, which serves to emphasize the importance of successful transactions by node *i* in the most recent trading period. Specifically, this coefficient indicates that the most recent trading periods will carry a higher weight in assessing the volume of successful transactions by node *i*, while the impact of earlier trading periods on node *i* will gradually diminish.

#### Definition 3

Energy type preference *G* is a reflection of environmental protection requirements. Within a trading cycle, a node’s energy type preference is defined as the energy type that appears most frequently in transactions. The reference values for different energy types are shown in Table 2. These reference values can be adjusted according to specific circumstances in practical applications. For instance, it is possible to raise the reference value for hydropower to encourage more nodes to choose hydroelectric energy resources, meeting environmental demands. Such adjustments can be made based on the actual situation to reflect various preferences and demands in the energy market.

**Table 2:** Energy type preference value.

Power generation type	Reference
Coal-fired power generation	0.3
Natural gas power generation	0.7
Nuclear power generation	0.8
Wind or solar power	1.0

#### Definition 4

Trustworthiness score *C*, in the S-PBFT consensus algorithm, is a crucial metric for nodes in the microgrid power trading network. The trustworthiness score of each node is determined by its transaction behavior in each trading cycle, and a unique global trustworthiness score is assigned to each node. In the microgrid electricity trading network, the global trustworthiness score of a node can be calculated using Eq. (6). This calculation process helps determine the level of trust each node has within the network, enabling them to participate more effectively in the consensus and leader election processes:

6 $$ Ci = \left( {T1iC1 + \cdots + TjiCj + \cdots + TniCn} \right)/n - 1. $$

*C*_*i*_ represents the trustworthiness score of node *i* within the entire network, with *T*_*ji*_ indicating node *j*’s reputation assessment of node *i*, and n denoting the total number of nodes in the network. Define the set *T* to represent node *I*’s credit assessment of other nodes in the microgrid power trading network, with $T = \left\{ {Ti1,Ti2. \cdots Tii - 1,\,\,Tii + 1,\, \cdots Tin} \right\}$ to initialize the set. If there were no transactions between node *i* and node* j* during a certain trading period* t*, then the value of *T*_*ji*_ is 0. However, if transactions occurred, *T*_*ji*_ can be calculated using Eq. (7):

7 $$ Tij = \frac{1}{M} \cdot \sum\limits_{m = 1}^{M} {s_{ij}^{m} } , $$

where *M* represents the number of transactions between node *i* and node *j* during trading period* t*, and $s_{ij}^{m}$ represents the transaction satisfaction of their *m*-th transaction.

#### Definition 5

Node election value *H* is used to assess the candidacy of node *i* when electing a leader node. The election value of node *i* can be calculated by comprehensively considering the previously mentioned election value factors and using Eq. (8). This election value helps determine whether node *i* is suitable to become a leader node, and its calculation method takes into account all relevant factors.

8 $$ \begin{gathered} H = B \cdot E \cdot G \cdot C \\ = \sum\limits_{r = 1}^{t} {\delta \cdot \frac{{b_{r}^{i} }}{{b_{r}^{\max } }}} \cdot \sum\limits_{r = 1}^{t} {\eta \cdot \sum\limits_{j = 1,j \ne i}^{n} {\sum\limits_{m = 1}^{m} {s_{ij}^{m} } } \cdot er_{ij}^{m} } \cdot G \cdot C. \\ \end{gathered} $$

Once the computation of *H* values for all nodes is completed, the *H* values of nodes within different consensus sets will be arranged in descending order. The node with the highest *H* value will be selected as the leader node. Subsequently, if a consensus node withdraws or becomes disconnected, the node with the second-highest H value will be elected as the new consensus node, and the consensus process will continue.

### S-PBFT dual-layer consensus process

**Figure 3:**
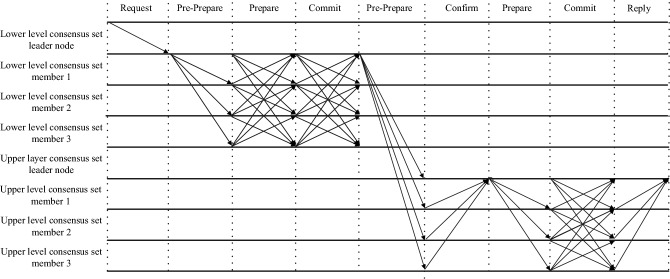
S-PBFT dual-layer consensus process.

In the blockchain network, participants are divided into K lower-level consensus sets. Before each consensus round, member nodes authenticate their identities using zero-knowledge proofs. Leader nodes are chosen based on the consensus set’s election strategy and publicly announce their identities within the network upon election. Other leader nodes verify the leader’s identity using a zero-knowledge proof authentication protocol. Finally, K leader nodes form the upper-level consensus set. In S-PBFT, lower-level consensus sets achieve local consensus, while the upper-level set achieves global consensus, creating the S-PBFT dual-layer consensus process. As depicted in Fig. 3, the leader node of the lower-level consensus set leads the remaining nodes to finalize the initial round of local consensus. Upon successful execution of local consensus, the leader node of the lower-level consensus set forwards the consensus result to the upper-level consensus set. Subsequently, it assumes the role of the leader node for the ongoing round of consensus in the upper-level, thereby completing the second round of global consensus.

It’s worth noting that within a blockchain network, when two nodes initiate a transaction, they first authenticate their identities using a zero-knowledge identity verification and key exchange algorithm. Subsequently, they obtain a shared key, denoted as K, to facilitate the transmission of transaction information and subsequent transaction processes. Records of the zero-knowledge identity verification process are stored on the blockchain. Consensus nodes must undergo an on-chain proof process to verify their identities before reaching consensus on the encrypted transaction information of the involved parties and proceeding with consensus. This ensures identity security and transaction trust within the electricity trading network.

#### Local consensus process

Block-producing node *m* is responsible for packing all transactions within a transaction cycle and sending a message*<<SPBFT-Request,h,d(b),t,d(ms)>σ*_*m*_*,b>*to the leader node *Lc* in the lower-level consensus set *Zc*, where S-PBFT-Request serves as the message identifier, *b* represents the block data, *h* denotes the current blockchain length, *d(b)* denotes the cryptographic hash of the block data, *t* represents the current timestamp, *d(ms)* represents the cryptographic hash of this message, and *σ*_*m*_ is the digital signature of node *m*. *Lc* authenticates the identity of *m* through an authentication protocol. After successful verification, *Lc* assembles the Pre-Prepare message with the format *<<SPBFT-PrePrepare,v,h,d(b),t,d(m)>σ*_*c*_*,b >*, where *v* represents the view number of the current round of consensus. The local consensus process primarily consists of three phases: pre-prepare, prepare, and commit.

##### Pre-prepare phase

*Lc* sends pre-prepare messages to the other nodes in the lower-level consensus set.

##### Prepare phase

In the lower-level consensus set, when regular nodes receive and successfully verify a Pre-prepare message, they store the corresponding block, generate Prepare messages based on this Pre-prepare message, and broadcast the Prepare messages to all other nodes except themselves. The format of a prepare message is *<<SPBFT-Prepare,v,h,d(b),t,d(m)>σ*_*i*_*>*. Nodes also receive Prepare messages from other nodes. If, within the consensus set, a node receives Prepare messages from more than 2/3 of the total nodes, and all these messages pass verification, the system proceeds to the commit phase.

##### Commit phase

The format of a commit message is *<<SPBFT-Commit,v,h,d(b),t,d(m)>σ*_*i*_*>*. Normal nodes in the lower-level consensus set send Commit messages to all nodes except themselves and receive Commit messages from other nodes. If nodes in the consensus set receive more than 1/3 of Commit messages and verify them successfully, the local consensus is completed.

#### Global consensus

After the completion of local consensus, the leader node representing the lower-level consensus set, *Lc*, will act as the leader node for the current round of global consensus, guiding other nodes within the upper-level consensus set to complete the global consensus process. Unlike local consensus, global consensus includes an additional Confirm phase, comprising four phases in total: pre-prepare, confirm, prepare, and commit.

##### Pre-prepare phase

*Lc* broadcasts the Pre-Prepare message in the upper-level consensus set with the following format: *<<SPBFT-PrePrepare,v,h,d(b),t,d(m)>σ*_*c*_*,b >*.

##### Confirm phase

When the remaining nodes in the upper-level consensus set receive the message and verify it successfully, they will save the block and generate Confirm messages based on the Pre-Prepare message. These Confirm messages are then sent to the leader node of the upper-level consensus set. The format of the Confirm message is *<<SPBFT-Comfirm,v,h,d(b),t,d(m)>σ*_*c*_* >*. At the same time, the leader node of the upper-level consensus set will receive Confirm messages sent by the other nodes. If the number of received Confirm messages exceeds 2/3 of the total number of nodes and these messages are verified, then the upper-level consensus set will enter the Prepare phase.

##### Prepare phase

After confirming the Confirm messages sent by the other nodes in the upper-level consensus set, the leader node of the upper-level consensus set will send Prepare messages to these nodes. The format of the Prepare message is *<<SPBFT-Prepare,v,h,d(b),t,d(m)>σ*_*c*_*>*.

##### Commit phase

The remaining nodes in the upper-level consensus set, upon receiving the Prepare message sent by the leader node and validating it, will broadcast Commit messages to all nodes except themselves and simultaneously receive Commit messages sent by other nodes. The format of the Commit message is *<<SPBFT-Commit,v,h,d(b),t,d(m)>σ*_*c*_* >*.If the nodes in the consensus set receive more than 2/3 of Commit messages and validate them, they proceed to the reply phase.

##### Reply phase

When the upper-level consensus set receives more than 1/3 of Reply messages, global consensus is achieved. Subsequently, each node adds the new block to its local blockchain and broadcasts the information about this new block to other nodes belonging to the same lower-level consensus set. The message format is *<<EPBFT-Result,v,h,d(b),t,d(m)>σ*_*c*_* >*.

After consensus is reached, the microgrid operator will update the information of various nodes based on the transaction data in the new block. Additionally, according to the consensus set leader election strategy, a new consensus leader node will be elected to prepare for the next round of consensus. This process ensures the continuous operation of the blockchain network and the ongoing consensus.

## Security analysis

### S-PBFT proof

In the S-PBFT consensus algorithm proposed in this paper, one round of S-PBFT consists of two key components: local consensus and global consensus. The local consensus phase employs the classical PBFT protocol, and as long as fewer than one-third of the nodes in the network are Byzantine nodes, the consensus process can be successfully completed. Therefore, this paper does not provide a detailed proof of local consensus, as it has already been extensively researched and validated. Next, this paper will focus on presenting the logical proof of global consensus. Global consensus is a secondary consensus process conducted after local consensus is achieved. Its purpose is to expedite information synchronization and updates, reduce system latency, and enhance the network’s scalability through the upper-level consensus set. If there are 100 consensus nodes in the blockchain network and they are divided into 20 consensus sets. Then there are a total of 20 nodes in the upper-level consensus set, assuming that there are 6 Byzantine nodes. Here is the specific logical proof:

In the Pre-Prepare phase, the leader node *c*_*u*_ in the lower-level consensus set broadcasts the message *<Pre-Prepare>c*_*u*_ to the upper-level consensus set, specifying that the nodes *c*_*i*_ in the upper-level consensus set receive the Pre-Prepare message as*<Pre-Prepare>c*_*i*_.

In the Confirm phase, nodes *c*_*i*_ within the upper-level consensus set send Confirm messages *<Confirm>c*_*i*_ to the upper-level consensus set leader node *c*_*0*_. In this context, it is established that if $\left\langle {Pre - Prepare} \right\rangle ci = \left\langle {Pre - Prepare} \right\rangle cu$, then $\left\langle {Confirm} \right\rangle ci = 1$; otherwise, $\left\langle {Confirm} \right\rangle ci = 0$. Considering that the total number of nodes in the consensus network is $N > 3f + 1$, hence the total number of nodes *n* within the upper-level consensus set is $n > 3f1 + 1$, where *f* represents the number of Byzantine nodes within the upper-level consensus set, From the assumption, we know that *n* = 20, *f*_*1*_ = 6. Therefore, the following relationship can be derived from this information:

9 $$ \sum\limits_{i = 1}^{19} {\left\langle {confirm} \right\rangle ci} \ge 2f1 + 1. $$

Next, the process move on to the Prepare phase. In the Prepare phase, the leader node *c*_*0*_ in the upper-level consensus set broadcasts the Prepare message *<Prepare>c*_*0*_ within the consensus set.

In the Commit phase, nodes in the upper-level consensus set exchange Commit messages with each other and receive Commit messages from other nodes, excluding themselves. The total sum of messages received by node *c*_*i*_ during the Commit phase can be represented as $M_{c0}^{commit}$:

10 $$ \begin{gathered} M_{ci}^{commit} = \left\langle {Commit} \right\rangle c0 + \left\langle {Commit} \right\rangle c1 + \cdots + \left\langle {Commit} \right\rangle ci - 1 \\ + \left\langle {Commit} \right\rangle ci + 1 + \cdots + \left\langle {Commit} \right\rangle cn. \\ \end{gathered} $$

It is specified in this paper that if *<Prepare>*_*ci*_ = *<Prepare>*_*c0*_, then *<Commit>*_*ci*_ = 1, thus:

11 $$ M_{ci}^{commit} \ge 2f1 + 1\left( {i = 1,2, \cdots ,20} \right). $$

In the Reply phase, since $M_{ci}^{commit} \ge 2f1 + 1$, it implies that in the Commit phase, at least *2f*_*1*_*+1* nodes can correctly process the messages. Therefore, the primary node will receive more than *2f*_*1*_*+1* Reply messages. Based on this observation, it can be concluded that the global consensus process in S-PBFT is secure when consensus is reached. This indicates that S-PBFT has sufficient fault tolerance to withstand errors from as many as f Byzantine nodes, ensuring the reliability of the consensus process.

### Defending against double spending attacks

Double spending attack is a security vulnerability and attack method in digital or cryptocurrency systems, with the aim of deceiving the system to enable the same unit of digital currency to be used multiple times, leading to the issue of duplicate payments. However, in the S-PBFT algorithm, double spending attack is impossible. According to the S-PBFT algorithm, each transaction must undergo a second confirmation by the upper-level consensus set after being confirmed by the lower-level consensus set. If an attacker node attempts to initiate two transactions, even though they may be recognized in the lower-level consensus, during the upper-level consensus process, nodes in the upper-level consensus set will verify the attacker node’s balance based on their own ledger. When an account balance cannot support both transactions simultaneously, the leader node will outright reject one of the transactions and flag the attacker node as a suspicious entity. Therefore, as long as the leader nodes in the upper-level consensus set are not Byzantine nodes, the S-PBFT algorithm effectively prevents double-spending attacks, thus ensuring the security and reliability of the digital currency system.

### Defending against denial of service (DoS) attacks

The goal of a denial of service attack is to congest the network with a large number of transaction requests, ultimately leading to network failure. However, S-PBFT employs an election mechanism to choose the leader nodes of the consensus set as a defense against this type of attack. In S-PBFT, the leader nodes for each round are generated through an election mechanism based on historical transaction records, and the identity of the leader nodes is unknown before the election. This means that even if attackers send a large number of invalid transaction requests to the system, the master nodes will not accept these requests. Therefore, S-PBFT is effective in resisting denial of service attacks, ensuring the security of the system.

### Defending against sybil attacks

The Sybil attack is a common form of attack where an attacker disrupts a peer-to-peer network or microgrid system by forging multiple identities or nodes. To defend against this type of attack, S-PBFT incorporates a key exchange algorithm based on zero-knowledge identity authentication, ensuring that each node or participant has a unique identity and can verify the identities of other nodes. In S-PBFT, each node corresponds to a real-world entity with the qualifications for electricity trading. Node identities are unique, and node digital signatures are generated using their private keys. Attackers do not possess the nodes’ private keys, making it impossible for them to forge node identities or message signatures. Therefore, in S-PBFT, no node can maintain multiple identities, effectively preventing sybil attacks. This ensures the system's trustworthiness and security.

### Defending against eclipse attacks

In an eclipse attack, attackers attempt to surround a target node with malicious nodes in order to establish connections only with these malicious nodes, isolating or blocking connections with honest nodes. However, the S-PBFT algorithm is effective in resisting such attacks. Based on the analysis in Sect. "S-PBFT Proof", we have demonstrated that S-PBFT possesses the same characteristics as PBFT, namely, its ability to tolerate up to 1/3 of Byzantine nodes among the total number of nodes. Therefore, only when an attacker can control more than 2/3 of the total number of nodes can they have an impact on the consensus results within the network and then launch a eclipse attack. However, in practical networks, such a situation is very rare. Therefore, the S-PBFT algorithm provides robust security, effectively preventing eclipse attacks.

## Performance analysis

In this study, we developed a blockchain simulation system on a personal computer equipped with a ThinkBook 16G4+ featuring an AMD Ryzen 7 6800H processor and 16.0 GB of memory. The system was implemented using the Go programming language. Within this simulation environment, we evaluated the performance of various consensus algorithms, namely PBFT, S-PBFT, P-PBFT, and C-PBFT^41^, by varying the number of nodes and conducting comparative assessments. Our experiments primarily focused on three crucial aspects: consensus latency, throughput, and communication overhead. Through these experiments and subsequent performance analyses, our objective was to attain a comprehensive understanding of how these algorithms perform within microgrid electricity trading scenarios.

### Communication overhead

Communication overhead refers to the amount of communication generated during the execution of a consensus algorithm by nodes, encompassing the total volume of messages and data exchanged between nodes. For consensus algorithms like PBFT that rely on information exchange, communication overhead becomes one of the key metrics for evaluating their performance. In a network with *N* nodes, during the Pre-Prepare phase, the primary node needs to broadcast Pre-Prepare messages to the other nodes in the network, resulting in *N-1* communications, as the primary node does not need to send this message to itself. During the Prepare phase, each node needs to broadcast Prepare messages to all nodes except itself, resulting in a communication count of *(N-1)*^2^, as each node has to send messages to all other nodes excluding itself. In the Commit phase, each node needs to broadcast Commit messages to other nodes, resulting in a communication count of *N(N-1)*, including sending Commit messages to itself. In summary, the communication overhead for one round of PBFT consensus can be calculated as:

12 $$ TPBFT = N - 1 + \left( {N - 1} \right)^{2} + N\left( {N - 1} \right) = 2N\left( {N - 1} \right), $$

If* N* nodes are divided into *k* lower-level consensus sets, with each lower-level consensus set containing *N/k* nodes, then the communication overhead for one round of S-PBFT consensus can be analyzed as follows:

In the local consensus, the leader node of the lower-level consensus set needs to broadcast Pre-Prepare messages to the ordinary nodes in that consensus set, with a communication count of *(N/k)-1*. After receiving the Pre-Prepare message, ordinary nodes validate its legitimacy and broadcast Prepare messages to all nodes in the lower-level consensus set, except for themselves, with a communication count of *(N/k-1)*^2^. Then, the ordinary node will receive Prepare messages from other nodes in the consensus set and proceed with the verification. Once verified, the ordinary node will send Commit messages to all nodes in the consensus set except itself, resulting in communication to *N/k (N/k-1)* nodes. In summary, the overall communication overhead for a round of local consensus in S-PBFT can be summarized as follows:

13 $$ \begin{gathered} T_{S - PBFT}^{l} = \left( {N/k} \right) - 1 + \left( {N/k - 1} \right)^{2} + N/k\left( {N/k - 1} \right) \\ = 2N/k\left( {N/k - 1} \right), \\ \end{gathered} $$

In the global consensus process of S-PBFT, the leader node of the lower-level consensus set broadcasts Pre-Prepare messages in the upper-level consensus set, ordinary nodes send Confirm messages to the leader node of the upper-level consensus set, the leader node in the upper-level consensus set broadcasts Prepare messages within its consensus set, and ordinary nodes send Reply messages to the leader node of the upper-level consensus set. The communication frequency for each of these actions is *k-1*, totaling *4(k-1)* communications. Next, ordinary nodes broadcast Commit messages to all nodes except themselves, with a communication frequency of *(k-1)*^2^. Finally, nodes in the upper-level consensus set broadcast the new block to their respective lower-level consensus sets, with a communication frequency of *N*. In summary, the total communication overhead for one round of S-PBFT global consensus can be summarized as follows:

14 $$ T_{S - PBFT}^{g} = 4\left( {k - 1} \right) + \left( {k - 1} \right)^{2} + N = \left( {k - 1} \right)\left( {k + 3} \right) + N. $$

By Eqs. (13) and (14), the total communication overhead of one round of S-PBFT consensus, $TS - PBFT$, can be calculated as:

15 $$ TS - PBFT = 2N/k\left( {N/k - 1} \right) + \left( {k - 1} \right)\left( {k + 3} \right) + N. $$

**Figure 4:**
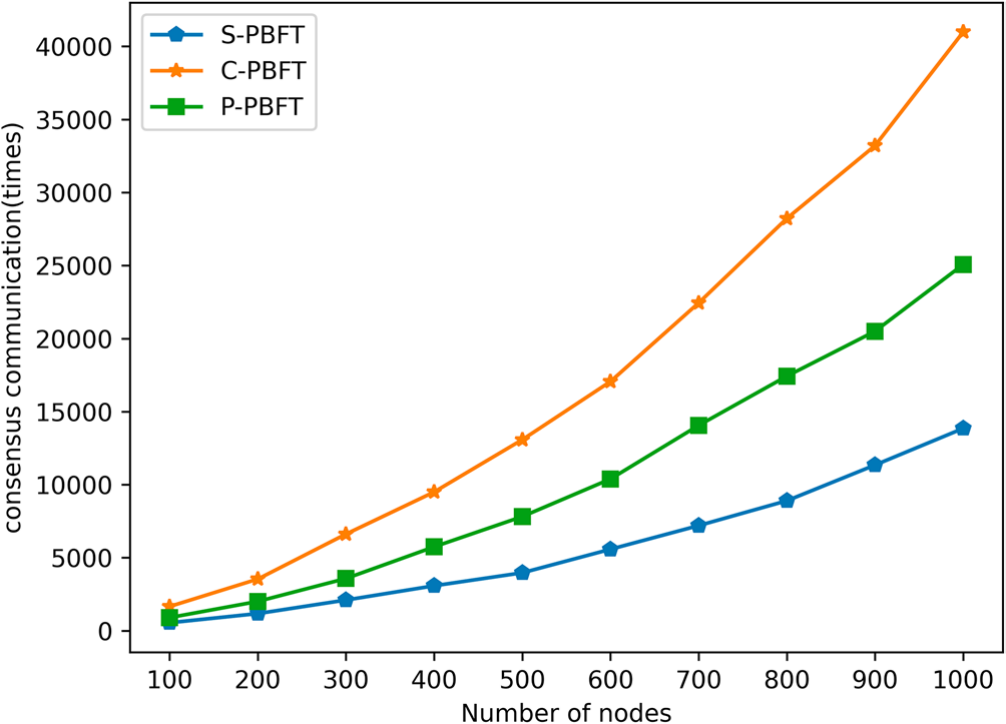
Consensus communications comparison among different algorithms.

In this experiment, we tested the single-round consensus communication volume of three consensus algorithms. At the beginning, we conducted tests with 100 nodes and 20 groups, and then, with the increase in the number of nodes, we added 100 nodes and 10 groups in each subsequent test. By observing the results displayed in Fig. 4**,** we can clearly see that with the increase in the number of nodes, the communication overhead of the three consensus algorithms also increases accordingly.

It is worth noting that the S-PBFT algorithm consistently excels in terms of communication overhead, remaining lower than the C-PBFT and P-PBFT algorithms, and its advantage becomes more pronounced as the number of nodes increases. For example, when the number of nodes increases to 500 with 60 groups, the S-PBFT algorithm reduces communication overhead by 47% compared to the P-PBFT algorithm and by 70% compared to the C-PBFT algorithm. Furthermore, when the number of nodes increases to 1000 with 110 groups, the S-PBFT algorithm reduces communication overhead by 48% compared to the P-PBFT algorithm and by 67% compared to the C-PBFT algorithm. These results clearly demonstrate that in networks of varying sizes, the S-PBFT algorithm can significantly reduce communication overhead, providing a noticeable improvement in blockchain consensus performance.

### Consensus delay

**Figure 5:**
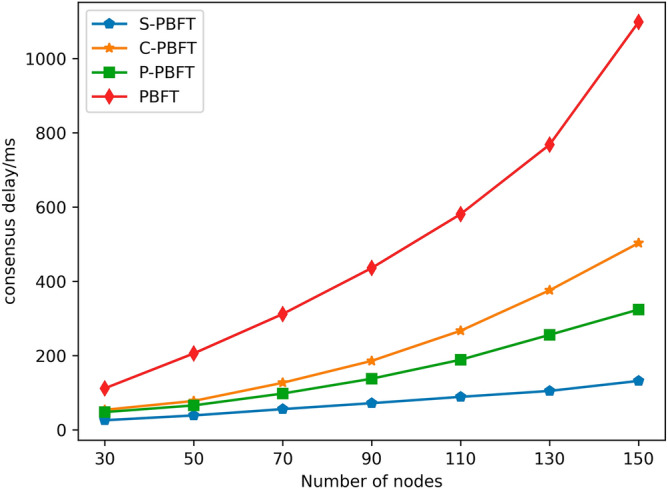
Consensus delay comparison among different algorithms.

Consensus latency is a key metric for evaluating the performance of consensus algorithms, representing the time it takes for a transaction to be completed from the moment a client sends a transaction request. A lower latency implies a faster execution speed of the consensus algorithm, higher consensus efficiency, allowing nodes within the network to reach consensus more quickly, thus enhancing the operational efficiency and security of the system. To evaluate the blockchain network with varying numbers of nodes, this paper chose a fixed group size of 5 and conducted 200 tests for different numbers of nodes. After every 20 experiments, the highest and lowest values were excluded, and the average was calculated to determine the experimental result. Based on the results from Fig. 5, it is evident that, at the same node count, the consensus latency of PBFT algorithm is notably higher than that of C-PBFT, P-PBFT, and S-PBFT algorithms. This is because the latter three employ a group consensus approach, which can significantly reduce communication overhead during the consensus process. As the number of nodes increases, the latency for all four algorithms also increases. However, it is worth noting that the growth rate of S-PBFT algorithm is the slowest. Particularly, when the number of nodes reaches 150, S-PBFT algorithm reduces consensus latency by 60% compared to P-PBFT algorithm and by 74% compared to C-PBFT algorithm. This indicates that the S-PBFT algorithm performs exceptionally well under high loads, effectively reducing consensus latency, and enhancing system performance.

### Throughput

TPS (transactions per second) is an important metric used to measure the speed and throughput of a system in processing transactions, especially in blockchain systems. The formula for calculating TPS is as follows:

16 $$ TPS = \frac{transactions\,\Delta t}{{\Delta t}}, $$

Where $transactions\Delta t$ is the number of transactions processed by the system within the time interval $\Delta t$, and $\Delta t$ represents the response time.

**Figure 6:**
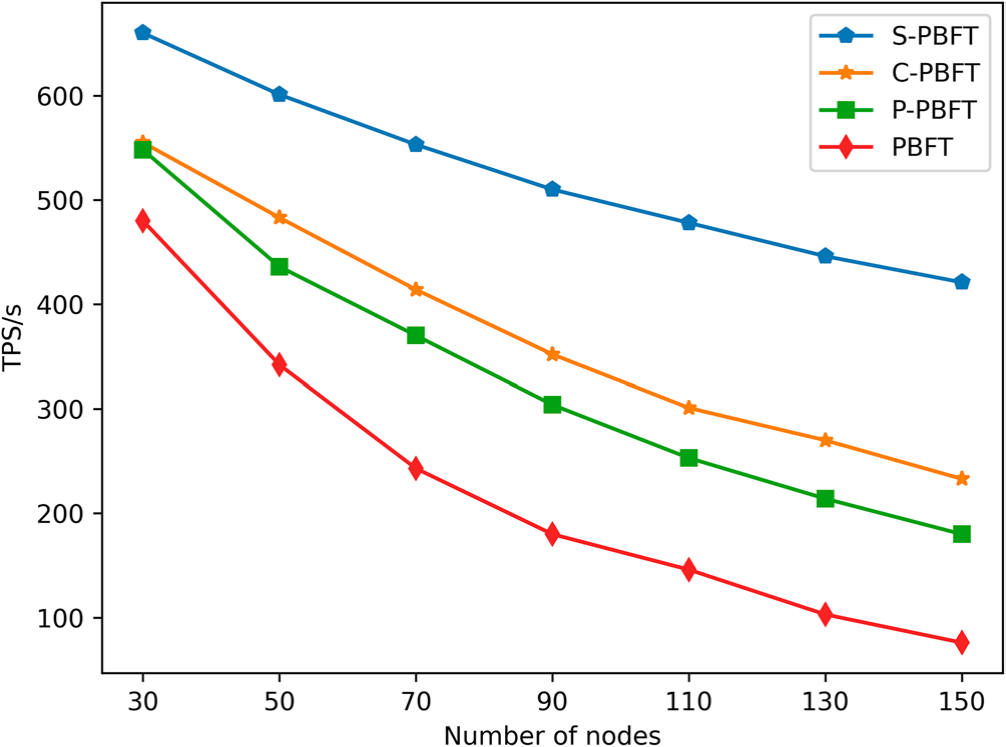
TPS comparison among different algorithms.

The TPS of PBFT-type algorithms is correlated with the number of nodes in the network. When there are many nodes, TPS decreases significantly due to the increased communication among nodes. In the throughput experiments, we maintained a consistent group size of 5 and conducted 200 tests with varying numbers of nodes to evaluate the Transactions Per Second (TPS) achieved per second. After every 20 experiments, the maximum and minimum values were excluded, and the average was taken as the experimental result. Based on Fig. 6, it can be observed that as the number of nodes increases, the system response time for PBFT, C-PBFT, P-PBFT, and S-PBFT gradually increases. It is worth noting that the system response time of PBFT decreases most significantly as the number of nodes increases from 30 to 90. While C-PBFT and P-PBFT have a slower rate of decrease compared to PBFT, they still experience significant drops when compared to S-PBFT. S-PBFT manages to maintain a relatively gradual decrease as the number of nodes increases incrementally. This is because S-PBFT adopts a two-tier consensus mechanism, where in each consensus round, it only requires preliminary consensus in the lower-level consensus set before obtaining secondary confirmation from the reliable upper-level consensus set. This mechanism not only ensures the accuracy of consensus but also reduces communication overhead among nodes, thus enhancing the throughput of the blockchain system. Furthermore, this characteristic of S-PBFT meets the demand for high-frequency transactions in microgrid power trading, thereby enhancing the system’s flexibility and efficiency.

## Conclusion

Blockchain technology holds great potential in the field of microgrid power trading, as it can improve transaction efficiency and security, reduce costs, and facilitate the integration of renewable energy sources. In this regard, an efficient and secure blockchain consensus algorithm for microgrid power trading, known as S-PBFT, is proposed. S-PBFT designs an authentication scheme that combines the Schnorr protocol with the Diffie-Hellman key exchange algorithm to achieve rapid authentication of node identities, enhancing system security. By employing a clustering algorithm, the nodes in the network are grouped into different clusters. Subsequently, based on specific election strategies, reliable nodes are chosen from each cluster to serve as the consensus set’s leader nodes. This ensures that the leader nodes in the consensus set possess good reputation and performance, thus enhancing the reliability of the consensus. Furthermore, the S-PBFT algorithm employs a two-tier consensus process, where the lower-level consensus set is responsible for initial consensus, while the upper-level consensus set provides secondary confirmation. This layered structure not only enhances the efficiency of consensus but also reduces inter-node communication, making the system more efficient. Security analysis indicates that the S-PBFT algorithm employs multiple layers of security measures to effectively defend against double-spending attacks, denial-of-service attacks, Sybil attacks, and eclipse attacks. Through mechanisms such as identity authentication, election strategies, and a two-layer consensus process, S-PBFT ensures the security and reliability of the system. Through extensive experimental testing, S-PBFT has demonstrated significant advantages in terms of communication overhead, consensus latency, and throughput. In the next phase, we will optimize the identity authentication protocol and the leader node election algorithm to further enhance the privacy protection and consensus security of the internal nodes in S-PBFT.

## Data Availability

The source code in this study is available on request from the corresponding author.
